# Associations between symptoms of attention-deficit hyperactivity disorder, socioeconomic status and asthma in children

**DOI:** 10.1038/s44184-024-00064-z

**Published:** 2024-04-16

**Authors:** Makiko Omura, Samuele Cortese, Marion Bailhache, Marie C. Navarro, Maria Melchior, Judith van der Waerden, Barbara Heude, Blandine de Lauzon-Guillain, Cédric Galera

**Affiliations:** 1 Department of Economics, Faculty of Economics, Meiji Gakuin University, Minato-ku, Tokyo, Japan; 2 Institut de Santé Publique d’Epidémiologie et de Développement, Université de Bordeaux, Bordeaux, France; 3 Center for Innovation in Mental Health, School of Psychology, Faculty of Environmental and Life Sciences, University of Southampton, Southampton, UK; 4 Clinical and Experimental Sciences (CNS and Psychiatry), Faculty of Medicine, University of Southampton, Southampton, UK; 5 Solent NHS Trust, Southampton, UK; 6 Hassenfeld Children’s Hospital at NYU Langone, New York University Child Study Center, New York City, NY USA; 7 DiMePRe-J-Department of Precision and Regenerative Medicine-Jonic Area, University “Aldo Moro”, Bari, Italy; 8 Centre Hospitalier Universitaire de Bordeaux—Urgences Pédiatriques, Bordeaux, France; 9 INSERM, Bordeaux Population Health Centre, UMR1219 Bordeaux, France; 10 Sorbonne Université, INSERM, Institut Pierre Louis d’Epidémiologie et de Santé Publique (IPLESP), UMR S 1136, Équipe de Recherche en Épidémiologie Sociale, F-75012 Paris, France; 11 Université Paris Cité and Université Sorbonne Paris Nord, INSERM, INRAE, Center for Research in Epidemiology and Statistics (CRESS), F-75004 Paris, France; 12 Centre Hospitalier Perrens, Bordeaux, France; 13 Research Unit on Children’s Psychosocial Maladjustment, Montreal, QC Canada

**Keywords:** Psychiatric disorders, Paediatric research

## Abstract

Socioeconomic status (SES) influences the risk of both physical diseases, such as asthma, and neurodevelopmental conditions, including attention-deficit/hyperactivity disorder (ADHD). Using Causal Mediation Analysis on French birth-cohort data, we found a causal pathway from SES to ADHD symptoms, in part mediated by asthma. An increase in family income at age 3 by one unit resulted in lower ADHD symptoms at age 5, by −0.37 [95% CI: −0.50, −0.24] SDQ-score-points, with additional −0.04 [95% CI: −0.08, −0.01] points reduction indirectly via asthma at age 3, both with statistical significance. Importantly, family income at age 3 exerted both direct and indirect (via asthma) negative effects on later ADHD symptoms with much higher magnitudes for the direct effect. Our findings underscore the importance of apprehending ADHD symptoms in the broader context of socioeconomic disparities, along with their comorbidities with asthma, potentially influencing public health interventions and clinical practice in managing ADHD.

## Introduction

More than 10% of children aged 4–18 experience mental health/neurodevelopmental problems, the most frequent being anxiety/depression (5.2–6.5%/1.3–2.6%), attention-deficit/hyperactivity disorder (ADHD) (3.4–3.7%), and conduct problems (4.6–5.7%)^1^. In terms of physical health, atopic diseases are amongst the most common conditions in children. While their prevalence varies across the globe, atopic diseases such as asthma, eczema (atopic dermatitis), allergic rhinitis, and food allergies are reported to affect about 20% of global population, and pose serious concerns especially among children^2,3^.

Both mental health and atopic symptoms can substantially reduce the health-related quality of life in patients as well as their families or caregivers. In the long-term, mental health and atopic symptoms can pose a considerable economic burden for affected individuals and the society by not only affecting the productivity of the caregivers but also prospective productivity of the children^2,4^. Despite their non-negligible impact, the exact aetiological mechanisms underlying mental and physical conditions are yet to be established. While mental health symptoms and immune hypersensitivity characterising atopic diseases seem to be distinct nosographic entities, various studies have found an association between them^5–11^. In particular, childhood asthma was shown as a potential predictor of later ADHD in a longitudinal analysis^12^. Studies have suggested a role of inflammatory mechanisms in explaining this association^5,13–15^. Other studies in twins identified a strong genetic component underlying the association between ADHD and asthma, while the role of environmental influences is still unclear^16–19^.

Current evidence shows a significant association between low SES and ADHD^20–23^ as well as between low SES and asthma^24–27^. Other studies also found a significant association between low SES and both ADHD and asthma^28,29^. However, the relationships among symptoms of ADHD, low SES and asthma are still unclear. Gaining insight into these relationships is relevant to inform preventive strategies for children at risk of ADHD symptoms. To this end, the present study aimed to analyse a potential causal pathway linking ADHD and asthma diseases using data from the French EDEN cohort study. We hypothesised a direct causal effect from low family SES to child ADHD and an additional indirect causal effect mediated by asthma.

## Methods

### Study sample

The EDEN cohort study was set up in 2003 to examine the pre- and early postnatal determinants of child health and development^30^. Study participation was proposed to all women visiting the university hospital prenatal clinic in Poitiers and Nancy, France between 2003 and 2006, before their 24th week of amenorrhoea. The exclusion criteria were multiple pregnancies, known diabetes before pregnancy, French illiteracy, or planning to move out of the region within the following 3 years. The study participants were not selected based on any of the diseases of interest in this study. Informed written consent was obtained from parent(s) at enrolment, and consent for the child to be in the study was obtained from both parents after birth. The study received approval from the ethics committee of Kremlin Bicêtre on 12th December 2002 and from the French data privacy institution, *Commission Nationale Informatique et Liberté*.

### Exposure: income at age 3

As child family income was measured in seven ranges by 700–800€ difference per month, we took the median value of the range in 1000€ (K€) unit as follows (ranges shown in parentheses), to be treated as a continuous variable: (1) 0.4 K€/m (<800€/m); (2) 1.15 K€/m (801–500€/m); (3) 1.9 K€/m (1501–2300€/m); (4) 2.65 K€/m (2301–3000€/m); (5) 3.4 K€/m (3001–3800€/m); (6) 4.15 K€/m (3801–4500€/m); (7) 4.9 K€/m (4500€/m<). Since there was no upper threshold for the highest category, we applied the same value range as others with additional 800€/m, i.e., 4500–5300€/m, which would be the most conservative threshold. We chose income rather than other SES proxies (e.g., parental education, occupation), because it was the most intervenable policy-wise target among other SES. Additionally, income is generally highly correlated with other SES indicators. The official threshold of poverty in France was 60% of median income, which amounted to 954€ per month for a single person in 2009^31^. Although the threshold age of 18 distinguishing adults and children in the EDEN cohort was different from the age threshold of 14 officially employed to calculate the average consumption unit (CU), the poverty line would be approximately in the third lowest income range for the Eden cohort, applying an average household composition of two adults and two children in the Eden cohort (CU = 1 (first adult) + 0.5 (second adult) + 0.3*2 (two children) = 2.1).

### Outcome: child ADHD symptoms at ages 5 and 8

We used the Strengths and Difficulties Questionnaire (SDQ)^32^ sub-scores for child inattention-hyperactivity (IH) to assess ADHD symptoms (SDQ-IH), measured at child’s age of 5 and 8 years. The IH problem scale comprised the following five items: (1) ‘restless, overactive’; (2) ‘constantly fidgeting or squirming’; (3) ‘easily distracted, concentration wanders’; (4) ‘thinks things out before acting (in opposite scale)’; and (5) ‘see tasks through to the end (in opposite scale)’. Each item scale is summed to a range of 0 to 10, with higher scores indicating higher difficulties. We used the continuous SDQ-IH score as an outcome, which was considered to represent the continuum risk of developing ADHD.

### Mediator: current asthma at age 3

Current asthma was defined as binary variable according to the MeDALL consortium criteria^33^. Children were considered as having current asthma if parents reported positive answer to at least two of the three following items: (1) ‘doctor-diagnosed asthma’; (2) ‘asthma medication in the past 12 months’; and (3) ‘wheezing in the past 12 months’.

### Pre-exposure covariates

The pre-exposure covariates were maternal age at birth (years), child sex assigned at birth (male vs. female), child birthweight (kg), and maternal smoking during pregnancy (average number of cigarettes per day). They were identified as potential confounders from available literature^14^ and from the directed acyclic graph depicting assumed relationships among variables (Supplementary Fig. 1). All of them were considered to be the mediator-outcome confounders. Only maternal age at birth was considered to additionally confound the exposure-mediator association, since younger maternal age could be associated with lower income and higher child asthma incidence^34^.

### Statistical analysis

Associations between variables were first examined using cross-sectional and longitudinal mixed-effects model accounting for repeated observations. With only SDQ-IH at age 3, 5 and 8 available with irregular intermittence, the longitudinal analysis assuming the same estimates across different ages might not capture intricate association between SDQ-IH and asthma. Thus, we focused on age-specific analysis yet ensuring temporary association. Four models were estimated with/without covariates to discern the underlying associations between the outcome (SDQ-IH), exposure (income), and mediator (asthma) variables to initially assess the appropriateness of the mediation model (here, ~ denotes ‘regressed on’: (Model 1) SDQ-IH~asthma (Y~M); (Model 2) SDQ-IH~income (Y~X); (Model 3) asthma~income (M~X). Additionally, we examined the opposite direction of pathway by estimating (Model 4) asthma ~ SDQ-IH (M~Y). All estimations included a medical centre dummy to account for any unmeasured geographical strata differences.

**Fig. 1:**
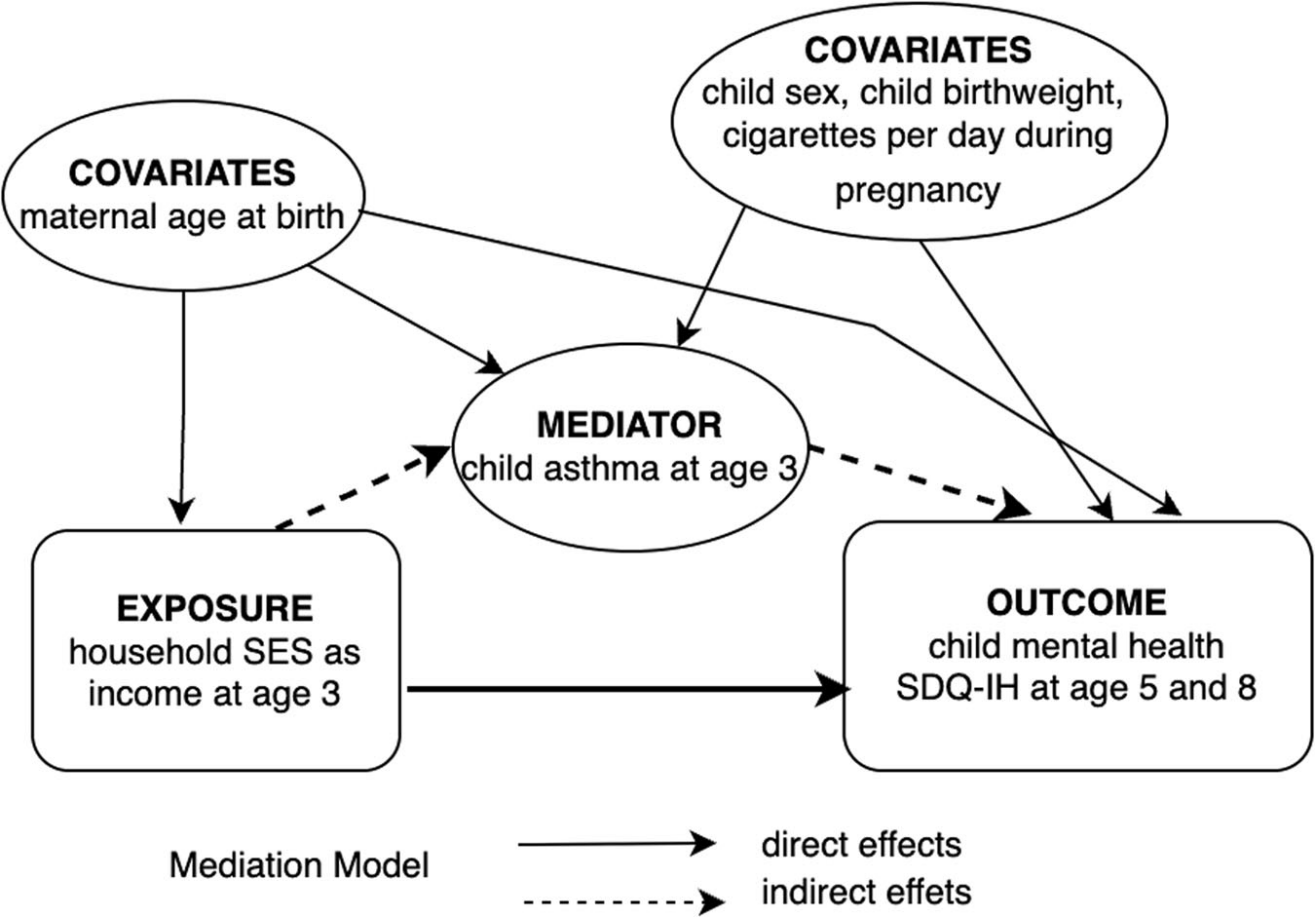
Mediation model with direct and indirect effects of income on SDQ-IH with covariates. The exposure, *income,* is modelled to exert the effect on the outcome, *IH problems,* directly and indirectly via the mediator, *asthma*. A potential exposure-mediator-outcome confounder, *maternal age at birth*, along with potential mediator-outcome confounders—namely, *sex*, *birthweight*, and *cigarette per day during pregnancy*—are controlled for.

For the main analysis, we conducted a causal mediation analysis (CMA) based on the natural effect or conditional mean model, within a nested counterfactual framework^35^ (see Supplementary Note 2 for further exposition). The causal inference based on the counterfactual approach allows the total causal effect to be decomposed into natural direct effect (NDE) and natural indirect effect (NIE) even in the presence of non-linearity and/or interaction effects^36^. The latest literature suggests the default inclusion of exposure-mediator interaction terms to capture the dynamics of mediaton^36^. Separating the interaction effect, the (pure) NDE is the causal effect of the exposure on the outcome, which is not mediated by any intermediate variables, and the (pure) NIE is the one mediated by one or more intermediate variables. To attain the necessary identification conditions for the CMA, i.e., implied certain independencies among exposure/mediator variables and potential outcomes as well as no omitted variables, we ensured the correct temporality of association and inclusion of identified confounders. Asthma as the mediator preceded the outcome. Income exposure and asthma were measured simultaneously, given the fact that income fluctuated annually despite showing high correlations across time. Moreover, earlier income was more highly correlated with an included pre-exposure covariate of maternal age at childbirth. The mediation model is depicted in Fig. 1, with income at age 3 as the exposure, SDQ-IH at age 5 and age 8 as the outcomes, and asthma at age 3 as the mediator.

Given that the missingness in our data was not related to any unobservable variables but was missing at random^37,38^, we conducted multiple imputation using multivariate imputation by chained equations (MICE) procedure (Supplementary Note 3). The imputation used the subjects who had at least one observed outcome, mediator and exposure. All model variables as well as auxiliary variables with correlations of about 0.4 and above with the model variables were included in the imputation. The number of data sets was decided to be 50, which was deemed more than sufficient as per the recommended rule of thumb^39^.

The multiple imputation and association analysis were conducted using Stata16. The CMA was conducted using R statistical software (ver.4.2.2) with the main package ‘medflex’ (ver.0.6–10)^35^.

## Results

### Study population

**Fig. 2:**
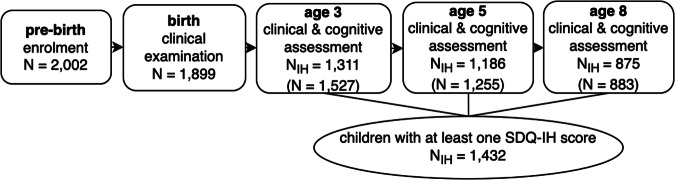
Participant flow chart and cohort with SDQ-IH information at ages 3, 5 and 8 years. The number of mother–child cohorts (*N*) is shown at each survey point. *N*_IH_ represents the number of cohorts in which the children underwent the clinical and cognitive assessment and for which the SDQ-IH data are available.

Among the 2002 women included in the EDEN cohort, 1527, 1255 and 883 mother–child pairs remained in the study when the child was 3, 5 and 8 years, respectively. Among them, 1311, 1186 and 875 children had their Strengths and Difficulties Questionnaire^32^ sub-scores for inattention-hyperactivity (SDQ-IH) collected for ADHD symptoms assessment, at age 3, 5 and 8, respectively (see Fig. 2 for participant flow chart). Consequently, 1432 children had at least one SDQ-IH data. Thirty percent of the women at baseline were pregnant for the first time. Women included in the EDEN cohort had a higher level of education, although they were similar in relation to other sociodemographic and birth-related characteristics, compared to the 2003 French National Perinatal Survey^30^.

### Descriptive statistics

Summary statistics of variables used in the main analysis with the sample of 1432 children based on multiple imputation are reported in Table 1. The SDQ-IH at age 5 and SDQ-IH at age 8 were 3.11 (SD: 2.4) and 3.2 (2.5), respectively. Asthma at age 3 had prevalence of 7%. A monthly family income at child’s age of 3, measured in seven categories but treated as continuous variable, had a mean level of 2.88 (SD: 1.08) in 1000€ unit. For the included pre-exposure covariates, sex had slightly higher representation of male (52%), and maternal age at birth ranged between 17 and 45, with an average age of 29.9. Mean birthweight was 3.29 kg. Average cigarettes per day during pregnancy had a mean value of 1.1 (SD: 2.7). The majority of mothers did not smoke during pregnancy (78%) while 3.3% of mothers smoked more than 10 cigarettes a day. Stratifying by sex, boys had higher mean SDQ-IH than girls by 24% (3.42 vs 2.76) at age 5 and by 32% (3.66 vs 2.78) at age 8. Boys also had slightly higher asthma prevalence compared to girls (8% vs 6%).

**Table 1:** Sample characteristics for all and by sex (EDEN cohort, No. = 1432; No. of males = 744, No. of females = 688)

	All (No. = 1432)		Male (No. = 744)		Female (No. = 688)		Missing rate
	Mean Freq.	(SD) %	Mean Freq.	(SD) %	Mean Freq.	(SD) %	
SDQ-IH at age 5* [0–10], mean (SD)	3.11	(2.38)	3.42	(2.45)	2.76	(2.26)	0.17
SDQ-IH at age 8* [0–10], mean (SD)	3.24	(2.51)	3.66	(2.58)	2.78	(2.35)	0.39
Asthma at age 3, No. (%)							0.08
*0. no*	1332	93%	683	92%	649	94%	
*1.* *yes*	101	7%	61	8%	39	6%	
Income at age 3 (median value of the range in 1000€), mean (SD), No. (%)	2.88	(1.08)	2.87	(1.07)	2.90	(1.10)	0.11
*1. 0.4 (<800€/m)*	28	2%	16	2%	12	2%	
*2. 1.15 (801–1500€/m)*	107	7%	49	7%	58	8%	
*3. 1.9 (1501–2300€/m)*	303	21%	164	22%	139	20%	
*4. 2.65 (2301–3000€/m)*	357	25%	191	26%	165	24%	
*5. 3.4 (3001–3800€/m)*	363	25%	191	26%	172	25%	
*6. 4.15 (3801–4500€/m)*	136	10%	61	8%	76	11%	
*7. 4.9 (4500€/m<)*	137	10%	72	10%	65	10%	
Sex, No. (%)							
*1. male*	744	52%					-
*2. female*	688	48%					-
Maternal age at childbirth (years), mean (SD)	29.93	(4.75)	29.94	(4.76)	29.92	(4.73)	-
Birthweight (kg), mean (SD)	3.29	(0.50)	3.35	(0.53)	3.23	(0.47)	-
Average cigarettes/day during pregnancy* [0–18.3], mean (SD)	1.09	(2.73)	1.03	(2.62)	1.16	(2.84)	0.02
Medical centre strata, No. (%)							
*1. Poitiers*	747	52%					-
*2. Nancy*	685	48%					-

The results shown are averaged values over 50 multiple imputed data set. Mean and standard deviation (SD) are reported for normally distributed variables. Non-normal distributed variables which are denoted by *. Note that non-normality does not pose an issue in terms of statistical analysis with a large sample size^48^. Frequency and percentage (%) are provided for categorical variables. While income is treated as continuous in the analysis, its ranges with frequencies and percentages (%) are presented as additional information. SDQ-IH stands for Strengths and Difficulties Questionnaire sub-scores for child inattention-hyperactivity.

### Association analysis

Model 1 looking at the relationship between SDQ-IH and preceding asthma suggested a positive association between SDQ-IH at age 5 and asthma at age 3 (0.51 [95% CI: −0.02; 1.04] (*p* = 0.061)) as well as SDQ-IH at age 8 and asthma at age 3 (0.64 [95% CI: 0.01; 1.27] (*p* = 0.045)) (Table 2). While the direct association between the outcome and the exposure was not required for mediation analysis, the negative association between SDQ-IH and income was highly robust and significant in Model 2. As shown in Table 3, a one-unit higher income at age 3 was associated with a lower SDQ-IH score, on average by 0.34 points [95% CI: −0.47; −0.21] (*p* < 0.001) at age 5 and 0.36 points [95% CI: −0.51; −0.22] (*p* < 0.001) at age 8. Regarding the association between asthma and income (Model 3) shown in the third column of Table 3, a unit increase in income at age 3 was on average associated with lower risk of asthma at age 3 by 0.76 [95% CI: 0.61; 0.94] (*p* = 0.011). The investigation of association between asthma and preceding SDQ-IH (Model 4) did not provide evidence of the opposite pathway (Supplementary Table 1).

**Table 2:** Association between asthma at age 3 and SDQ-IH at age 5 and 8 (Model 1) (Eden cohort, No. = 1432)

	SDQ-IH at age 5		SDQ-IH at age 8	
	Unadjusted	Fully adjusted	Unadjusted	Fully adjusted
	*β* coefficient, 95% CI, *p* value	*β* coefficient, 95% CI, *p* value	*β* coefficient, 95% CI, *p* value	*β* coefficient, 95% CI, *p* value
Asthma at age 3	0.72	0.51	0.82	0.64
	[0.17, 1.26]	[−0.02, 1.04]	[0.19, 1.44]	[0.01, 1.27]
	[0.010]	[0.061]	[0.011]	[0.045]

The 95% confidence intervals and *p* values are in brackets. Fully adjusted for sex, maternal age at childbirth, child birthweight, cigarettes per day during pregnancy and medical centre. SDQ-IH stands for Strengths and Difficulties Questionnaire sub-scores for child inattention-hyperactivity. Estimated using 50 imputed datasets.

**Table 3:** Association between family income at age 3 and SDQ-IH at age 5 and 8 (Model 2), and the association between family income at age 3 and asthma at age 3 and (Model 3) (Eden cohort, No. = 1432)

	SDQ-IH at age 5		SDQ-IH at age 8		Asthma at age 3	
	Unadjusted	Fully adjusted	Unadjusted	Fully adjusted	Unadjusted	Fully adjusted
	*β* coefficient, 95% CI, *p* value	*β* coefficient, 95% CI, *p* value	*β* coefficient, 95% CI, *p* value	*β* coefficient, 95% CI, *p* value	Odds Ratio, 95% CI, *p* value	Odds Ratio, 95% CI, *p* value
Income at age 3	−0.47	−0.34	−0.42	−0.36	0.72	0.76
	[−0.60, −0.35]	[−0.47, −0.21]	[−0.55, −0.28]	[−0.51, −0.22]	[0.59, 0.89]	[0.61, 0.94]
	[<0.001]	[<0.001]	[<0.001]	[<0.001]	[0.001]	[0.011]

The figures in asthma at age 3 model are in odds-ratio (OR). Income at age 3 is treated as continuous. The 95% confidence intervals and *p* values are in brackets. Fully adjusted for sex, maternal age at childbirth, child birthweight, cigarettes per day during pregnancy and medical centre. SDQ-IH stands for Strengths and Difficulties Questionnaire sub-scores for child inattention-hyperactivity. Estimated using 50 imputed datasets.

### Causal mediation analysis

The CMA estimated effects of income at age 3 on SDQ-IH at age 5 and age 8 mediated by asthma at age 3 are reported in Table 4, providing estimates for the NDE, NIE, interaction effect (NDE*NIE), and total effect, along with the proportion mediated. The total effect is the sum of the NDE, NIE and mediated interactive effect. The NDE estimate was −0.37 [−0.50; −0.24] (*p* < 0.001), the NIE estimate was −0.04 [−0.08; −0.01] (*p* = 0.026), the interaction effect was 0.01 [0.001; 0.02] (*p* < 0.030) with total effect of −0.40 [−0.54, −0.26] (*p* < 0.001) for SDQ-IH at age 5. A non-zero interaction effect implied that the income effect on SDQ-IH varied by the asthma status and vice-versa. Therefore, a unit increase in income was associated with lower SDQ-IH score on average by 0.40 points. The estimated figures were similar for SDQ-IH at age 8, although with lager CI for NIE. The sex-stratified CMA results for SDQ-IH at age 5 in Table 5 exhibit that both NDE (−0.45 [−0.64; −0.26] (*p* < 0.001)) and NIE (−0.08 [−0.16; −0.004] (*p* *=* 0.054)) were higher in magnitudes for boys. While NDE (−0.29 [−0.48; −0.11] (*p* = 0.002)) was also detected in girls, no NIE was detected, suggesting the mediation effect only among boys. The estimation with original data (complete case analysis) provided in Supplementary Table 2 reveals consistent and stronger results compared with those obtained with multiple imputed data. An estimate of the opposite direction treating asthma as the outcome and SDQ-IH as the mediator is given in Supplementary Table 3 which shows no mediation effect. A sensitivity analysis with additional covariates is provided in Supplementary Table 4 with Supplementary Note 1, which also gives the estimation results of all covariates in the CMA.

**Table 4:** Causal mediation analysis: Natural direct and indirect effects of income at age 3 on SDQ-IH at age 5 and SDQ-IH at age 8 mediated by asthma at age 3 (Eden cohort, No. = 1432)

	SDQ-IH at age 5			SDQ-IH at age 8		
	*β* coefficient	95% CI	*p* value	*β* coefficient	95% CI	*p* value
NDE	−0.37	[−0.50, −0.24]	<0.001	−0.39	[−0.53, −0.24]	<0.001
NIE	−0.04	[−0.08, −0.01]	0.026	−0.04	[−0.08, 0.01]	0.084
Interaction (NDE*NIE)	0.01	[0.001, 0.02]	0.030	0.01	[−0.003, 0.02]	0.117
Total effect	−0.40	[−0.54, −0.26]	<0.001	−0.41	[−0.58, −0.25]	<0.001
Proportion mediated	0.11			0.09		

Outcome = SDQ-IH at age 5 and 8 (continuous); Exposure = income at age 3 (continuous); Mediator = asthma at age 3 (binary). CI are calculated using robust standard errors based on the sandwich estimator. CI calculated using bootstrap method produced almost identical results. Fully adjusted for sex, maternal age at childbirth, child birthweight, cigarettes per day during pregnancy and medical centre. The counterfactual values were derived using imputation-based approach in *Medflex* R-package. Total effect = (pure) NDE + (pure) NIE + interaction term. Estimated using 50 imputed datasets.

**Table 5:** Causal mediation analysis: sex-stratified natural direct and indirect effects of income at age 3 on SDQ-IH at age 5 mediated by asthma at age 3 (Eden cohort, No. of males = 744, No. of females = 688)

	SDQ-IH at age 5 male			SDQ-IH at age 5 female		
	*β* coefficient	95% CI	*p* value	*β* coefficient	95% CI	*p* value
NDE	−0.45	[−0.64, −0.26]	<0.001	−0.29	[−0.48, −0.11]	0.002
NIE	−0.08	[−0.16, −0.004]	0.041	0.004	[−0.03, 0.04]	0.830
Interaction (NDE*NIE)	0.03	[0.000, 0.05]	0.054	−0.001	[−0.01, 0.01]	0.876
Total effect	−0.50	[−0.71, −0.29]	<0.001	−0.29	[−0.48, −0.10]	0.003
Proportion mediated	0.16			−0.01		

Outcome = SDQ-IH at age 5 for male and female (continuous); Exposure = income at age 3 (continuous); Mediator = asthma at age 3 (binary). CI is calculated using robust standard errors based on the sandwich estimator. CI calculated using the bootstrap method produced almost identical results. Fully adjusted for sex, maternal age at childbirth, child birthweight, cigarettes per day during pregnancy and medical centre. The counterfactual values were derived using imputation-based approach in *Medflex* R-package. Total effect = (pure) NDE + (pure) NIE + interaction term. Estimated using 50 imputed datasets.

## Discussion

In this French birth cohort EDEN, we found evidence of a longitudinal pathway from low income to ADHD symptoms, partially mediated by asthma. The positive association found between child asthma and ADHD is in line with previous research in the US, Canada, Denmark, and South Korea^5,6,12,28,40^, quantitatively summarised in systematic reviews/meta-analyses^7,10,11,14,41^. A possible explanation for this association might be that both conditions involve inflammation and immune system dysregulation. The allergic inflammation characterising asthma might affect the prefrontal cortex region and neurotransmitter system implicated in ADHD^5,13,14,42^. Additionally, common genetic vulnerability might be involved. Nonetheless, while some authors have pointed to a shared genetic vulnerability for ADHD and asthma^18,43^, others have been more cautious in such claim^15^. Taking advantage of the longitudinal design, our data showed that the direction of the association is likely to be from asthma to ADHD, rather than the other way around (Supplementary Table 1 and Supplementary Table 3) as reported in previous literature^17^. This suggests not only the possible benefit for integrated treatment approaches for children with asthma and ADHD, but also that children with early-age asthma to be monitored for the possible later development of ADHD.

The negative associations between SES and child ADHD symptoms are in line with previous studies conducted in the US, Norway, South Korea, the UK, Australia, Canada, the Netherlands and Sweden^20–23,44^. Likewise, the negative association between SES and child asthma is in accordance with existing evidence shown for children in the UK, Australia, Germany, the US^24,25,27,45,46^, as well as a meta-analysis across high-income countries^26^.

While studies in some countries may suggest the possibility of low-income families having limited access to healthcare resources, resulting in a lack of appropriate diagnosis and treatment for asthma and/or ADHD^25^, this is unlikely in the French context with universal healthcare. This also makes the reverse causality from child health status to family income less likely as the French medical care system covers most of the direct cost. Earlier SES disadvantages have been indicated to predict higher level of inflammation that may lead to asthma/ADHD^47^. Overall, the finding of a negative association between asthma/ADHD and income highlights the need for increased attention and support for children and families of low SES affected by asthma/ADHD.

Notably, we found that income exerted negative effect directly on ADHD and indirectly via asthma, with much higher magnitudes for the direct effect. Our CMA results suggested that an increase of monthly income by 1000€ was associated with a lower SDQ-IH score on average by 0.40 points, and about 10% would be mediated by asthma. While to our knowledge no previous research investigated the possible causal pathway from SES to ADHD mediated by asthma, there are few studies assessing the effect of SES on ADHD and asthma^28,29^. Our findings are coherent with the study^29^ reporting that children with asthma with lower SES, but not in higher, were prone to ADHD symptoms, and also with the study^28^ where low SES was identified as an additional risk to the associated asthma and ADHD. Overall, this evidence points to the benefit of extended support and attention for lower SES children with early-age asthma, when aiming at reducing the risk of developing ADHD at later stage.

To our knowledge, this is the first study to explore the causal pathway from SES to ADHD via asthma taking the advantage of longitudinal data and the mediation approach based on the counterfactual framework. We thereby offered novel insights into the intricate relationship between SES, asthma, and ADHD. An additional strength is the choice of confounders controlled for. Of note, these confounders were significant themselves, which could partly explain the complex relationships (Supplementary Table 4). For instance, being male, lower birthweight, younger maternal age and more cigarettes per day were all associated with higher risk of ADHD, confirming the past findings. Our use of multiple imputed data mitigated possible bias caused by attrition and missing data. The complete case analysis in the CMA displayed higher coefficient magnitudes and higher statistical significance for both age 5 and age 8 (Supplementary Table 2).

In terms of limitations, although we exploited the advantage of longitudinal data in our analysis, some challenges remain, such as nonoptimal measurement times, omitted variables or paths, and misspecification of the mediator. For instance, we cannot rule out the possibility of other, more relevant mediators such as parental psychopathology and adversity incidents^22^, given the relatively small mediation effect of asthma found in our analysis. In terms of the data, we only had binary asthma values, and having their severity measure could have been more informative for our analysis^26^. Likewise, we cannot exclude the possibly of a family incurring the indirect opportunity cost of caring for the child with asthma/ADHD symptoms, thus negatively impacting the family income, despite the French medical care system covering most of the direct cost. Additionally, we did not have data on participants’ precise income nor household composition to enable per consumption unit income calculation to obtain more accurate effect estimates. It would be of interest to examine also actual environmental triggers, such as pollens, housedust, moulds, pets, food and diet, or genetic or immunological factors for asthma/ADHD, including the parental history of these symptoms. However, some of environmental triggers, as well as parental genetic or immunological factors, could be closely related to family income. While the CMA required identification conditions, we could not completely rule out the possibility of omitted confounders. Nonetheless, we conducted a sensitivity analysis with additional available covariates which ensured the robustness of our findings (Supplementary Table 4).

Our study highlights the need for extended support and attention for lower SES children, and particularly for those with early-age asthma, so as to reduce the risk of developing ADHD at later stage. From a public health perspective, reinforcing preventive measures targeting more vulnerable populations in terms of SES may help reduce private and social burdens of mental and physical health. This study used a French birth cohort, and further investigation using other cohort is required to apply the findings generally. Our results provide the rationale for studies testing to what extent early treatment of asthma can decrease the risk of future ADHD. Further research is also needed to establish the pathophysiological pathway given that our study highlights the importance of apprehending mental health problems in the broader context of socioeconomic disparities, and with its comorbidities in terms of physical health.

### Supplementary information

Supplementary_Information_out

## Data Availability

The data underlying the findings are available on reasonable request and with permission from the EDEN Steering Committee to be contacted at etude.eden@inserm.fr.
