# Supplementary material for: Associations between symptoms of attention-deficit hyperactivity disorder, socioeconomic status and asthma in children
Source: Npj Ment Health Res. 2024 Apr 16;3:22. doi: 10.1038/s44184-024-00064-z (PMC11021421; doi:10.1038/s44184-024-00064-z)
Supplement: Supplementary file 1 — Supplementary_Information_out [file 44184_2024_64_MOESM1_ESM.pdf]

SUPPLEMENTARY INFORMATION

Supplementary Table 1 Opposite Association between SDQ-IH at age 3 and asthma at age 5, and between SDQ-IH at age 5 and asthma at age 8 (Model 4) (Eden cohort, N=1,432)

	asthma_5			asthma_8	
	unadjusted	fully adjusted		unadjusted	fully adjusted
SDQ-HI at age 3	1.03	1.00	SDQ-HI at age 5	0.99	0.95
	[0.95, 1.12]	[0.91, 1.09]		[0.89, 1.10]	[0.85, 1.06]
	[0.488]	[0.946]		[0.853]	[0.336]

Notes: Figures in odds-ratio (OR). The 95% confidence-intervals and p-values in brackets. Fully adjusted for sex, maternal age at childbirth, child birthweight, cigarettes per day during pregnancy and medical centre. Estimated using 50 imputed datasets.

Supplementary Table 2 The effect of income at age 3 on SDQ-IH at age 5 and 8 mediated by asthma at age 3 with original data (complete case) estimated by the CMA (Eden cohort; N at age5=1,009, N at age8=785)

	SDQ-IH at age 5			SDQ-IHHI at age 8		
	Estimate	95 % CI	p-value	Estimate	95 % CI	p-value
natural direct effects	-0.44	[-0.60, -0.28]	<0.001	-0.47	[-0.482, -0.231]	<0.001
natural indirect effects	-0.08	[-0.14, -0.02]	0.008	-0.08	[-0.469, -0.010]	0.023
interaction (NDE*NIE)	0.02	[0.01, 0.04]	0.010	0.02	[0.001, 0.024]	0.029
total effects	-0.50	[-0.67, -0.32]	<0.001	-0.53	[-0.73, -0.33]	<0.001
proportion mediated	0.16			0.16		

Notes: Outcome = SDQ-ADHD at age 5 and 8 (continuous); Exposure = income at age 3 (continuous); Mediator = asthma at age 3 (binary). CI are calculated using robust standard errors based on the sandwich estimator. CI calculated using bootstrap method produces almost identical results. The counterfactual values are derived using imputation-based approach in *Medflex* R-package. Total effects = (pure) NDE + (pure) NIE + interaction term. Fully adjusted for sex, maternal age at childbirth, child birthweight, cigarettes per day during pregnancy and medical centre

Supplementary Table 3 Opposite Causal Effect: the effect of income at age 3 on asthma at age 5 and 8 mediated by SDQ-IH at age 3 estimated by the CMA (Eden cohort; N=1,432)

	Asthma at age 5 (OR)			Asthma at age 8 (OR)		
	Estimate	95 % CI	p-value	Estimate	95 % CI	p-value
natural direct effects	0.79	[0.63, 0.99]	0.044	0.88	[0.67, 1.16]	0.380
natural indirect effects	0.91	[0.80, 1.03]	0.124	1.05	[0.92, 1.17]	0.486
interaction (NDE*NIE)	1.04	[1.00, 1.08]	0.073	1.00	[0.96, 1.04]	0.916
total effects	0.75			0.93		

Notes: Outcome = Asthma at age 5 and 8 (binary); Exposure = income at age 3 (continuous); Mediator = SDQ-IH at age 3 (continuous). Estimates are in odds-ratio (OR). CI are calculated using robust standard errors based on the sandwich estimator. CI calculated using bootstrap method produces almost identical results. The counterfactual values are derived using imputation-based approach in *Medflex* R-package. Total effects = (pure) NDE + (pure) NIE + interaction term. Fully adjusted for sex, maternal age at childbirth, child birthweight, cigarettes per day during pregnancy and medical centre. Estimated using 50 imputed datasets.

SUPPLEMENTARY INFORMATION

Supplementary Note 1 Sensitivity Analysis

Since the identification assumptions cannot be directly tested, several methods of sensitivity analysis have been proposed. One proposed method requires to hypothesise the effect of the unmeasured confounder on the outcome and the mediator,[1] which can be perplexing itself. Another approach which conceptualises the unmeasured confounder based on the correlated errors terms of the mediator regression and the outcome regression models[2,3] has not been extended to MI data. Given the fact that the *Medflex* package did not provide a sensitivity analysis, we conducted a sensitivity analysis with additional available covariates to assess the robustness of our findings in view of possible omitted variables. In addition to the original fully-adjusted CMA model, we estimated the CMA with other available covariates (z) which were: mother's education; father's education; parental cohabitation status; parental marital status; mother's housing status during pregnancy; cognitively stimulating activity scores at age 2 and at age 3; and eczema at age 2. The estimated results showed highly robust estimates for the direct effect, indirect effects, interaction term and other included covariate in the original model.

Supplementary Table 4 Sensitivity Analysis of the CMA for SDQ-ADHD at age 5 with additional covariates (Eden cohort, N=1,432)

	SDQ-ADHD at age 5		SDQ-ADHD at age 5		SDQ-ADHD at age 5		SDQ-ADHD at age 5		SDQ-ADHD at age 5	
	original		z = mother's education[(a)]		z = father's education[(a)]		z = live with father at age 3[(b)]		z1 = marital status at age 3_category2[(c)] z2 = marital status at age 3_category3[(c)]	
	estimate	p-value	estimate	p-value	estimate	p-value	estimate	p-value	estimate	p-value
natural direct effect	-0.37	<0.001	-0.27	0.001	-0.23	0.002	-0.35	<0.001	-0.34	<0.001
natural indirect effect	-0.04	0.026	-0.05	0.033	-0.05	0.023	-0.04	0.049	-0.04	0.028
sex (female)	-0.65	<0.001	-0.66	<0.001	-0.65	<0.001	-0.66	<0.001	-0.66	<0.001
maternal age at birth	-0.05	<0.001	-0.05	<0.001	-0.05	0.001	-0.05	0.001	-0.05	0.001
birthweight	-0.27	0.032	-0.27	0.033	-0.26	0.037	-0.27	0.031	-0.27	0.032
pregnancy smoking	0.07	0.011	0.06	0.027	0.06	0.025	0.07	0.011	0.06	0.024
medical centre	-0.24	0.068	-0.22	0.095	-0.20	0.117	-0.25	0.056	-0.21	0.100
interaction (NDE*NIE)	0.01	0.030	0.01	0.032	0.01	0.029	0.01	0.051	0.01	0.030
add. covariate z			-0.08	0.010	-0.13	<0.001	-0.34	0.316	-0.01	0.981

add. covariate z									-0.37	0.046

	SDQ-ADHD at age 5		SDQ-ADHD at age 5		SDQ-ADHD at age 5		SDQ-ADHD at age 5		SDQ-ADHD at age 5	
	z = maternal housing d)		z = mother's profession €		z = cognitively stimulating activities at age 2(f)		z = cognitively stimulating activities at age3(f)		z = eczema at age 2(g)	
	estimate	p-value	estimate	p-value	estimate	p-value	estimate	p-value	estimate	p-value
natural direct effect	-0.37	<0.001	-0.33	<0.001	-0.39	<0.001	-0.38	<0.001	-0.37	<0.001
natural indirect effect	-0.04	0.028	-0.05	0.028	-0.04	0.027	-0.04	0.029	-0.04	0.028
sex (female)	-0.65	<0.001	-0.65	<0.001	-0.66	<0.001	-0.69	<0.001	-0.65	<0.001
maternal age at birth	-0.05	0.001	-0.05	0.001	-0.05	<0.001	-0.05	<0.001	-0.05	0.001
birthweight	-0.27	0.031	-0.26	0.037	-0.27	0.029	-0.27	0.029	-0.27	0.033
pregnancy smoking	0.07	0.012	0.06	0.016	0.07	0.011	0.06	0.015	0.07	0.012
medical centre	-0.23	0.068	-0.22	0.085	-0.20	0.122	-0.17	0.185	-0.24	0.065
interaction (NDE*NIE)	0.01	0.030	0.01	0.033	0.01	0.031	0.01	0.031	0.01	0.032
additional covariate z	-0.03	0.773	-0.05	0.206	0.08	<0.001	0.10	<0.001	0.21	0.402
additional covariate z										

Note: Original controls: sex; maternal age at childbirth in years; birthweight in kg; number of cigarettes during pregnancy; medical centre dummy. Following additional covariates were considered as z: (a) education years for mother and father; (b) binary variable for living with the father at age 1 and age 3; (c) marital status: reference category = single/divorced/ separated; category2 = PACS/widow/concubine; category3 = officially married; (d) housing during pregnancy ordered: 1 = owner; 2 = tenant; 3 = family/friend's house; 4 = free house; (e) mother's profession in the order of average income; (f) cognitive stimulating activities = sum of each score ranging from 1 (never or hardly ever) to 5 (every day or almost every day) for the following activities for/with the child; singing, reading, playing, going for a walk, ball games; eczema as binary according to MeDALL (Pinart et al., 2014).[4] The counterfactual values are derived using imputation-based approach in *Medflex* R-package. Estimated using 50 imputed datasets.

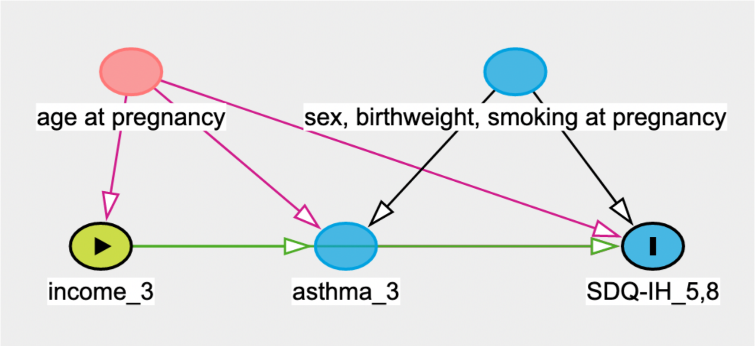

Supplementary Figure 1 Directed Acyclic Graphs (DAG) for income, asthma and SDQ-IH
The directed acyclic graph (DAG) portrays the causal diagram reflecting the mediation hypothesis along with the identification assumption.[5] In particular, the causal association is depicted with income at age 3 as the exposure, SDQ-IH at age 5 or age 8 as the outcome, asthma at age 3 as the mediator. Identified confounders are: maternal age at pregnancy as for the associations between exposure–mediator, exposure–outcome, and mediator–outcome; sex, birthweight and smoking at pregnancy as confounders for the association between mediator–outcome.

Supplementary Note 2 Causal Mediation Analysis (CMA): Counterfactual Framework and Effect Decomposition

The CMA is based on the counterfactual approach introduced in the causal inference literature, allowing the estimation of the causal effects defined as comparisons of potential outcomes.[5–7] Within this counterfactual framework, Robins and Greenland (1992)[8] and Pearl (2001)[6] proposed a method of estimating the causal effects and decomposing the total effect into direct and indirect effects, even with the presence of an interaction term or nonlinearity.

As we cannot observe both potential outcomes for any person, what we estimate is the average causal effect for a population. The causal inference approach rests on two assumptions, namely the consistency assumption and the composition assumption. Setting Y as the outcome, A as the exposure, M as the mediator, the consistency assumption states that when A = a, the potential outcomes Y(a) and M (a) equal the actual observed outcomes Y and M, respectively, i.e., Y(a) = Y and M(a) = M. The composition assumption states that when M(a) = m, then Y(a) = Y(a, m). This essentially means that, the potential outcome Y(a) that would be observed when A = a would match the potential outcome Y(a,m) that would be observed when A = a and M = m, setting the value of the intermediate variable M for exposure A = a as M = m. The effects defined in the causal inference literature are described below, mainly adopting from VanderWeele's (2015)[1] exposition. Conditioned on the pre-exposure covariates, C=c, the total effects (TE) are decomposed into three parts, pure direct effect (PDE), pure indirect effect (PIE), and mediated interaction effect (INT):

Total effects (TE): $E[TE(a, a^*)|c]=E[Y(a)-Y(a^*)|c] = PDE + PIE + INT$
Pure direct effect (PDE): $E[PDE(a, a^*)|c]=E[Y(a, M(a^*))-Y(a^*, M(a^*))|c]$
Pure indirect effect (PIE): $E[PIE(a, a^*)|c]=E[Y(a^*, M(a))-Y(a^*, M(a^*))|c]$
Mediated interaction effect (INT): $E[INT(a, a^*)|c]=E[Y(a, M(a))-Y(a^*, M(a))-Y(a, M(a^*))+Y(a^*, M(a^*))|c]$

The PDE expresses how much the outcome would change on average if the exposure changed from the reference level A = a∗ to level A = a, with the mediator for each individual fixed at the natural level that it would have taken at exposure level A =a*. On the other hand, the PIE captures how much the outcome would change on average if the exposure were fixed at level A = a∗, but the mediator was changed from the natural level it would take if A = a∗ to the level that would have taken at exposure level A = a. By combining PDE and INT, we obtain the total direct effect (TDE), and by combining PIE and INT, we obtain the total indirect effect (TIE), which can be defined as follows:

Total direct effect (TDE): $E[TDE(a, a^*)|c]=E[Y(a, M(a))-Y(a^*, M(a))|c] = PDE + INT$
Total indirect effect (TIE): $E[TIE(a, a^*)|c]=E[Y(a, M(a))-Y(a, M(a^*))|c] = PIE + INT$

SUPPLEMENTARY INFORMATION

The TDE depicts how much the outcome would change on average if the exposure changed from the reference level $A = a*$ to level $A = a$, but the mediator for each individual was fixed at the natural level that it would have taken at exposure level $A = a$. The TIE measures how much the outcome would change on average if the exposure were fixed at level $A = a$, but the mediator was changed from the level it would take if $A = a^*$ to the level it would take if $A = a$.

While natural direct effect (NDE) and natural indirect effect (NIE) can refer to TDE and TIE, respectively, we refer NDE as natural PDE and NDE as natural PIE, separately treating the exposure-mediator interaction effect, INT.

Because the counterfactual is never observed, it is necessary for the following four conditions to be met in order for these effects to be identified:[1,6,9]

(i) $Y(a, m)$ for $\forall$ a, m $\perp\perp A|C$

(ii) $Y(a, m)$ for $\forall$ a, m $\perp\perp M|\{ A, C\}$

(iii) $M(a)$ for $\forall a \perp\perp A|C$

(iv) $Y(a, m)$ for $\forall a, a^*, m \perp\perp M(a^*)|C$

Each condition above states that, given the observed pre-exposure covariates: (i) there is no unmeasured confounding between the exposure-outcome relationship; (ii) there is no unmeasured confounding between the mediator-outcome relationship; (iii) there is no unmeasured confounding between the exposure-mediator relationship; (iv) there should be no variable that is affected by the exposure that confounds the mediator-outcome relationship. These identification conditions essentially signify no-unmeasured-confounding, which cannot be directly tested. A set of sensitivity analysis is provided to examine the robustness of the CMA results in presence of additional covariates (Supplementary Note1).

Supplementary Note 3 Multiple Imputation

Our data suggested attrition biases in which lower SES families attrite at each stage, and more children with asthma attrite at age 5. In addition, there was also some intermittent missingness. The fact that the missingness was explained by the observed lower SES and asthma and not related to our outcome SDQ-measures, it could be inferred that the missingness was not related to any unobservable variables but was missing at random (MAR).[10,11] Given that our data could be considered as MAR, it made sense to apply multiple imputation (MI).

We applied MI using chained equations (MICE) which was a principled method of addressing arbitrary missing-value patterns and which could deal with numerical and categorical variables. In order to ensure consistency, all variables included in the analytical model were included in the imputation model.[12,13] The dataset for MI was limited to those children with at least one observation on the outcome, exposure, and mediator. All model variables and additional covariates used for sensitivity analysis well as auxiliary variables with correlations of about 0.4 and above, namely, SDQ-comportment at age 3, 5 and 8, income measured at pre-birth (24th week of amenorrhoea) and age 1, 2, 4 and 8, mother's profession and father's profession, were included in the imputation. The missing value percentages of the variables are given in eTable.1. For all continuous variables, we used the predictive mean matching imputation method as the normality of underlying model was not assured. Ten nearest neighbours were included in the set of possible donors from which an imputed value was randomly drawn. For binary variables, logistic regression was applied. The MICE procedure was performed using *mi* programme in STATA16 and 50 datasets were imputed. Based on the recommended rule of thumb,[14] this number of datasets was deemed sufficient

Supplementary Table 5 Complete, Missing and Imputed Observations for the Multiple Imputation of the Eden Cohort Data (50 data sets)

	Complete, Missing and Imputed Observations per MI dataset				
Variable	Complete	Missing	(% Missing)	Imputed	Total
SDQ-IH at age 5	1186	246	(0.17)	246	1432
SDQ-IH at age 8	875	557	(0.39)	557	1432
asthma at age 3	1311	121	(0.08)	121	1432
income at age 3	1281	151	(0.11)	151	1432
sex	1432	0	-	0	1432
maternal age at childbirth	1432	0	-	0	1432
birthweight	1432	0	-	0	1432
maternal smoking	1400	32	(0.02)	32	1432
medical centre	1432	0	-	0	1432
	(additional covariates for sensitivity analysis)				
mother's education	1426	6	-	6	1432
father's education	1321	111	(0.08)	111	1432

live with father at age 3	1316	116	(0.08)	116	1432
marital status at age 3	1315	117	(0.08)	117	1432
property	1426	6	(0.004)	6	1432
mother's profession	1344	88	(0.06)	88	1432
cognitively stimulating activity at age 2	1277	155	(155)	155	1432
cognitively stimulating activity at age 3	1310	122	(122)	122	1432
eczema at age 2	1262	170	(170)	170	1432

Note: Complete + Incomplete = Total; Imputed is the minimum across m of the number of filled-in observations. Observations without any SDQ-IH are excluded from the original data to be imputed. See eTable4 note for variable description. Imputation done with STATA 16.

SUPPLEMENTARY INFORMATION

Supplementary References

1. VanderWeele TJ. Explanation in causal inference: methods for mediation and interaction. New York: Oxford University Press; 2015. 706 p.
2. Imai K, Keele L, Tingley D. A general approach to causal mediation analysis. Psychol Methods. 2010;15(4):309–34.
3. Imai K, Keele L, Tingley D, Yamamoto T. Causal Mediation Analysis Using R. In: Vinod HD, editor. Advances in Social Science Research Using R [Internet]. New York, NY: Springer New York; 2010 [cited 2022 May 1]. p. 129–54. (Lecture Notes in Statistics; vol. 196). Available from: http://link.springer.com/10.1007/978-1-4419-1764-5_8
4. Pinart M, Benet M, Annesi-Maesano I, von Berg A, Berdel D, Carlsen KCL, et al. Comorbidity of eczema, rhinitis, and asthma in IgE-sensitised and non-IgE-sensitised children in MeDALL: a population-based cohort study. Lancet Respir Med. 2014 Feb 1;2(2):131–40.
5. Pearl J. Causal diagrams for empirical research. Biometrika. 1995;82(4):669–88.
6. Pearl J. Direct and Indirect Effects. Proc Seventeenth Conf Uncertain Artif Intell 411–420 San Franc Morgan Kaufmann. 2001;10.
7. Rubin DB. Causal Inference Using Potential Outcomes: Design, Modeling, Decisions. J Am Stat Assoc. 2005;100(469):322–31.
8. Robins JM, Greenland S. Identifiability and Exchangeability for Direct and Indirect Effects. Epidemiology. 1992;3(2):143–55.
9. Vanderweele TJ, Vansteelandt S. Conceptual issues concerning mediation, interventions and composition. Stat Interface. 2009;2(4):457–68.
10. Azur MJ, Stuart EA, Frangakis C, Leaf PJ. Multiple imputation by chained equations: what is it and how does it work?: Multiple imputation by chained equations. Int J Methods Psychiatr Res. 2011 Mar;20(1):40–9.
11. Little RJA, Rubin DB. Statistical analysis with missing data. Third edition. Hoboken, NJ: Wiley; 2020. 1 p. (Wiley series in probability and statistics).
12. Schafer JL, Graham JW. Missing data: Our view of the state of the art. Psychol Methods. 2002 Jun;7(2):147–77.
13. Young R, Johnson DR. Handling Missing Values in Longitudinal Panel Data With Multiple Imputation. J Marriage Fam. 2015 Feb;77(1):277–94.
14. White IR, Royston P, Wood AM. Multiple imputation using chained equations: Issues and guidance for practice. Stat Med. 2011;30(4):377–99.
